# Antibody drugs conjugates in non–small cell lung cancer: current status and challenges

**DOI:** 10.1093/oncolo/oyaf331

**Published:** 2025-10-06

**Authors:** Arjun Syal, May-Lucie Meyer, Kenneth Angelino, Noah Osei, Jorge E Gomez, Triparna Sen, Fred R Hirsch

**Affiliations:** Department of Internal Medicine, Icahn School of Medicine at Mount Sinai, Mount Sinai Morningside and West, New York, NY 10019, United States; Center for Thoracic Oncology, Tisch Cancer Institute and Icahn School of Medicine at Mount Sinai, New York, NY 10029, United States; Department of Oncology, Centre Hospitalier Universitaire Vaudois (CHUV), 1011 Lausanne, Switzerland; Center for Thoracic Oncology, Tisch Cancer Institute and Icahn School of Medicine at Mount Sinai, New York, NY 10029, United States; Center for Thoracic Oncology, Tisch Cancer Institute and Icahn School of Medicine at Mount Sinai, New York, NY 10029, United States; Center for Thoracic Oncology, Tisch Cancer Institute and Icahn School of Medicine at Mount Sinai, New York, NY 10029, United States; Department of Internal Medicine, The Ohio State University, Columbus, OH 43210, United States; The Ohio State University Comprehensive Cancer Center—Arthur G James Cancer Hospital and Richard J Solove Research Institute, The Ohio State University, Columbus, OH 43210, United States; Center for Thoracic Oncology, Tisch Cancer Institute and Icahn School of Medicine at Mount Sinai, New York, NY 10029, United States

**Keywords:** non–small cell lung cancer, NSCLC, thoracic oncology, biomarkers, antibody–drug conjugate, ADC

## Abstract

**Background:**

Antibody–drug conjugates (ADCs) are an emerging class of therapeutics that combine the specificity of monoclonal antibodies with cytotoxic or immune-stimulatory payloads. In non–small cell lung cancer (NSCLC), they offer a novel strategy with potential in both first-line therapy and in cases to overcome resistance to existing targeted and immune-based therapies.

**Objective:**

To review the clinical development, efficacy, safety, biomarker strategies, and emerging targets of ADCs in NSCLC, with a focus on implications for practice and ongoing challenges.

**Methods:**

We conducted a comprehensive literature review of published trials, conference abstracts, and press releases evaluating ADCs in NSCLC, with attention to target antigens, clinical trial outcomes, and biomarker approaches.

**Results:**

ADCs targeting *HER2, TROP2,* and *c-MET* have received regulatory approval in NSCLC, with demonstrated efficacy—particularly in biomarker-selected populations. Bispecific HER3/epidermal growth factor receptor (EGFR)-directed ADCs have shown encouraging activity in early phase studies, with ongoing trials expected to clarify durability and optimal patient selection. Other targets such as ITGB6, B7-H3, and AXL have shown early signals of efficacy. Predictive biomarkers vary in reliability, and mutation, amplification, or protein expression do not uniformly predict response. Toxicity and acquired resistance remain key challenges; improved diagnostics may enhance patient selection.

**Conclusion:**

ADCs are poised to reshape the therapeutic landscape of NSCLC. Their success will hinge on refining biomarker strategies, managing toxicity, and integrating resistance-mitigating approaches such as bispecific constructs or rational combinations. As research advances, ADCs may become essential components of personalized therapy across a range of molecular and histologic NSCLC subtypes.

Implications for practice Antibody–drug conjugates (ADCs) are an emerging treatment class in non–small cell lung cancer (NSCLC), showing efficacy across diverse molecular subtypes. This review summarizes key clinical trials, biomarker considerations, and safety profiles of ADCs currently approved or in development. As these agents gain regulatory traction, particularly in biomarker-selected populations, a practical understanding of their indications, limitations, and ongoing trials will be essential for clinicians integrating ADCs into the treatment of patients with NSCLC.

## Introduction

Lung cancer remains the leading cause of cancer-related mortality worldwide, accounting for over 1.8 million deaths annually.^1^ Non–small cell lung cancer (NSCLC), which comprises approximately 85% of cases, represents a biologically heterogeneous group with diverse histologic and molecular profiles. Although tobacco remains the main risk factor, a subset—especially younger, female, and never-smokers—harbors oncogenic driver alterations such as epidermal growth factor receptor (EGFR) mutations and anaplastic lymphoma kinase (ALK) rearrangements.^2^ These cases underscore the importance of molecularly guided treatment.

Over the past 2 decades, the NSCLC therapeutic landscape has shifted. Targeted therapies and immunotherapy are now foundational in advanced NSCLC and are increasingly used in earlier stages. However, resistance remains nearly universal, highlighting the need for strategies that address on-target and bypass mechanisms.^3^

**Figure 1.**
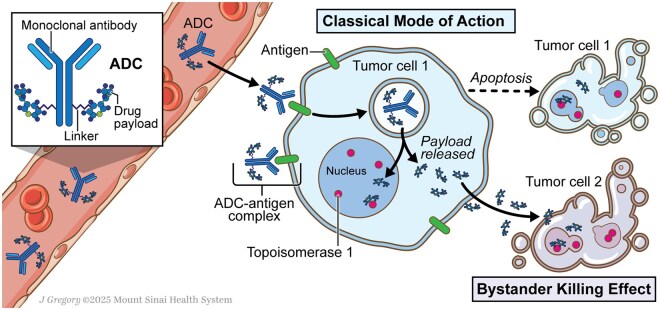
Structure and mechanism of action of antibody–drug conjugates (ADCs): The basic structure of an ADC and its mechanistic features including internalization into the cell, release of payload, bystander killing effect, and anti-tumor immunity via effector cells.

Antibody–drug conjugates (ADCs) have emerged as a promising therapeutic class that combines the target specificity of monoclonal antibodies with cytotoxic or immune-stimulatory payloads. By delivering these payloads to tumor antigens, ADCs increase tumor kill and reduce off-target toxicity (Figure 1).^4^ The 2022 approval of trastuzumab deruxtecan (T-DXd) for *HER2*-mutant NSCLC marked a critical milestone in this space, establishing ADCs as a treatment modality in thoracic oncology.^5^ Additional ADCs are in development across diverse targets and settings.

This review examines ADCs approved or in development for NSCLC, with emphasis on pivotal trials, emerging agents, and challenges including resistance and toxicity. We provide interpretation of key data and discuss future directions that may influence how ADCs are integrated into clinical practice.

## Materials and methods

This review focuses on ADCs approved or in clinical development for NSCLC. Sources were identified via PubMed using “antibody-drug conjugates,” “ADC,” and “NSCLC,” along with ClinicalTrials.gov, oncology abstracts (eg, ASCO, ESMO, and WCLC), and reference lists. Recent press releases were also included.

### ADCs: Current status of ADCs in NSCLC treatment

Initial development of ADCs in NSCLC focused on biomarker-defined populations, particularly tumors harboring *HER2* mutations. However, the presence of a targetable genomic alteration does not always predict ADC response, likely due to the multifactorial biology of these agents driven by target, trafficking, payload, and bystander biology.^6^^,^^7^ As a result, identifying an optimal ADC target requires more than genomic profiling; it demands a deeper understanding of the role of protein expression, internalization dynamics, and the tumor microenvironment.

These complexities have prompted the investigation of ADCs in broader, biomarker-agnostic populations, sometimes without prior tissue-based target confirmation.^8^ Whether efficacy is driven by genomic alterations, antigen expression, or other biological factors remains unsolved and critical for patient selection.^9^

**Figure 2.**
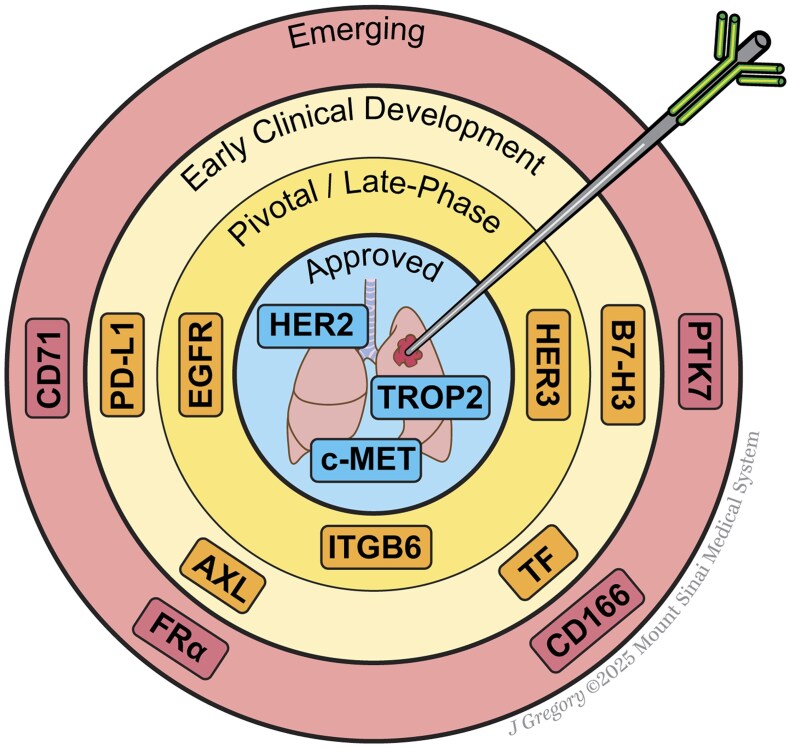
Concentric-circle schematic of ADC targets in NSCLC. The center circle shows targets with FDA-approved ADCs (HER2, TROP2, and c-MET). The next annulus highlights pivotal and late-phase development (EGFR, ITGB6, and HER3). The following ring depicts agents in early clinical development (PD-L1, AXL, TF, and B7-H3). The outermost circle includes emerging and translational targets (CD71, FRα, CD166, and PTK7). Abbreviations: ADC, antibody–drug conjugate; B7-H3, B7 homolog 3; CD166, activated leukocyte cell adhesion molecule; CD71, transferrin receptor-1; EGFR, epidermal growth factor receptor; FRα, folate receptor α; HER2, human epidermal growth factor receptor 2; ITGB6, integrin β6; NSCLC, non–small cell lung cancer; PD-L1, programmed death-ligand 1; PTK7, protein tyrosine kinase 7; TF, tissue factor; TROP2, trophoblast cell-surface antigen 2.

The following sections highlight the most clinically advanced ADCs in NSCLC, organized by target antigen. Key efficacy and safety data are summarized in Table 1. Additionally, Figure 2 summarizes ADC targets in NSCLC, spanning approved agents, those in clinical development, and emerging exploratory targets.

**Table 1.** Select late-phase and clinically informative antibody–drug conjugate (ADC) trials in non–small cell lung cancer (NSCLC).

ADC name	Target	Trial (phase)	Biomarker context	Population	Objective response rate (%); progression-free survival (months)	Grade ≥3 treatment-related adverse events (%)	Approval status
**Trastuzumab emtansine (T-DM1)**	*HER2*	Phase 2 basket trial	HER2 mutation (IHC ranged from 0 to 2+)	Pre-treated HER2m NSCLC	44; 5	6	Not approved for NSCLC
**Trastuzumab deruxtecan (T-DXd)**	*HER2*	Destiny-Lung01 (2)	HER2 mutation	Pre-treated HER2m NSCLC	55; 8.2	46	Accelerated approval
**Trastuzumab deruxtecan (T-DXd)**	*HER2*	Destiny-Lung01	HER2 overexpression IHC 2+/3+	Pre-treated HER2 NSCLC	25; 5.4	74	Accelerated approval
**Trastuzumab deruxtecan (T-DXd)**	*HER2*	Destiny-Lung02 (5.4 mg/kg) (2)	HER2 mutation	Pre-treated HER2m NSCLC	49; 9.9	39	Accelerated approval
**Telisotuzumab vedotin (Teliso-V)**	*c-MET*	LUMINOSITY (2)	IHC 3+ in ≥50% tumor cells (EGFR WT only)	Pre-treated c-MET high, EGFR WT NSCLC	35; 5.5	30	Accelerated approval
**Datopotamab deruxtecan (Dato-DXd)**	*TROP2*	TROPION-Lung05 (3)	EGFR mutation	Pre-treated advanced EGFRm NSCLC	44; 5.8	28^a^	Accelerated approval
**Sacituzumab govitecan (SG)**	*TROP2*	EVOKE-02 (2)	No selection; reported by PD-L1 TPS	First-line metastatic NSCLC, PD-L1 ≥ 50% (SG + pembro)	67-73^b^	NR	Investigation under evoke-03

This table highlights ADCs evaluated in advanced or metastatic NSCLC across pivotal, accelerated-approval–supporting, or practice-informing trials. Reported outcomes include objective response rate (ORR), progression-free survival (PFS), and grade ≥3 treatment-related adverse events (TRAEs), unless otherwise specified. FDA approval status is specific to the NSCLC indication unless noted. NR, not reported in the corresponding publication or abstract.

Destiny-Lung01 includes 2 separate cohorts:

– *HER2*-mutant: ORR 55%, PFS 8.2 months.

– *HER2* IHC 2+/3+: ORR 25%, PFS 5.4 months.

**T-DM1** data derived from a phase 2 basket trial (NCT02675829) in HER2-mutant NSCLC; HER2 expression ranged from IHC 0 to 2+.

Abbreviation: IHC, immunohistochemistry.

a Grade ≥3 TRAE of 28% for datopotamab deruxtecan reflects the full TROPION-Lung05 study population, not limited to the *EGFR*-mutant subgroup.

b ORR of 67%-73% in EVOKE-02 reflects PD-L1 ≥ 50% squamous and non–squamous cohorts; lower ORR was observed in PD-L1 < 50% groups. EVOKE-03 is ongoing.

### HER2

Human epidermal growth factor receptor 2 (HER2) is a receptor tyrosine kinase that can be altered in NSCLC through activating mutations, amplification, or protein overexpression. Among these, the exon 20 mutation is the most clinically established biomarker for HER2-directed ADCs.^10-12^

Trastuzumab emtansine (T-DM1) was first evaluated in NSCLC in a phase 2 basket trial of heavily pretreated patients with *HER2*-mutant lung adenocarcinomas.^13^ The trial showed an objective response rate (ORR) of 44%. Responses were lower when patients were selected by immunohistochemistry (IHC) alone versus activating mutations. Very little hematologic side effects were seen. Two patients with concurrent *HER2* amplification and extracellular domain mutations (S310F and S335C) achieved partial response (PR) and stable disease (SD), respectively, suggesting a role for amplification in modulating ADC sensitivity. These findings support *HER2* mutation as a more reliable biomarker while acknowledging overexpression and amplification may still have therapeutic relevance.

The phase 2 DESTINY-Lung01 trial enrolled patients with mostly pretreated *HER2*-mutant NSCLC and reported an ORR of 55%, median progression-free survival (PFS) of 8.2 months, and overall survival (OS) of 17.8 months with T-DXd at 6.4 mg/kg.^14^ Interstitial lung disease (ILD) occurred in 26% of patients, including 2 deaths, with 46% experiencing any grade ≥3 TRAEs, most commonly neutropenia (19%) and anemia (10%). A separate cohort of HER2 IHC 2/3+ patients (regardless of mutation) showed a lower ORR of 24.5%, supporting the greater predictive value of *HER2* mutations over IHC expression.^15^ This discrepancy may reflect differences in HER2 biology between NSCLC and other tumor types where IHC is more predictive, such as breast or gastric cancer.^16^

DESTINY-Lung02 was a randomized phase 2 trial evaluating T-DXd at 5.4 versus 6.4 mg/kg in previously treated *HER2*-mutant NSCLC. The 5.4 mg/kg cohort showed a confirmed ORR of 49%, with comparable efficacy and improved safety: ILD rates were 5.9% versus 8.0%, grade ≥3 TRAEs occurred in 39.0% versus 58.0%, and rates of grade ≥3 neutropenia and anemia were 18.8% versus 36.0% and 10.9% versus 16.0%, respectively. These findings supported selection of the 5.4 mg/kg dose and led to FDA accelerated approval in 2022, reinforcing the importance of optimizing tolerability as ADCs move into earlier treatment settings.^17^

Although NSCLC was largely excluded from the DESTINY-PanTumor02 trial,^18^ pooled results from PanTumor02, DESTINY-Lung01, and DESTINY-CRC02 led to a 2024 tissue-agnostic accelerated approval of T-DXd for HER2-overexpressing tumors.^19^ A phase 3 trial (DESTINY-Lung04) is ongoing in *HER2*-mutant NSCLC. These findings highlight *HER2* mutations as the most reliable biomarker, while *HER2* overexpression and amplification may still warrant further study. Optimizing selection will likely require integrating genomic, proteomic, and histopathologic data.

### c-MET

c-MET is a transmembrane receptor tyrosine kinase activated by hepatocyte growth factor (HGF). In NSCLC, c-MET dysregulation includes overexpression (up to 70%), amplification (0.7%-21%), and exon 14 skipping mutations (2%-3%).^20^^,^^21^ Amplification may contribute to acquired resistance in EGFR-mutant disease, while exon 14 mutations are targetable with MET inhibitors.^21^ In contrast, c-MET protein overexpression, measured by IHC, has historically lacked effective targeted therapies. However, with the efficacy demonstrated by telisotuzumab vedotin (Teliso-V), overexpression has now emerged as a clinically relevant and therapeutically actionable biomarker, uniquely suited to ADC-based strategies.^22-24^

Teliso-V is an ADC that links an anti-c-MET monoclonal antibody to monomethyl auristatin E (MMAE). The phase 2 LUMINOSITY trial employed a 2-stage, 3-cohort design to evaluate *Teliso-V* in patients with c-MET protein–overexpressing advanced NSCLC.^24^ Patients were stratified into: (1) nonsquamous, EGFR wild-type; (2) nonsquamous, EGFR-mutant; and (3) squamous histology. Only the nonsquamous, EGFR wild-type cohort advanced beyond stage I. All patients had c-MET protein overexpression as defined by IHC (SP44 assay, Roche), with further stratification into high (IHC ≥50%) and intermediate (IHC 25%-49%) expression levels.

Among EGFR wild-type patients, Teliso-V demonstrated ORR of 28.6% (95% confidence interval [CI], 21.7-36.2), increasing to 34.6% (95% CI, 24.2-46.2) in those with high expression with a median PFS of 5.5 months (95% CI, 4.1-8.3). These findings led to breakthrough therapy designation and, on May 14, 2025, accelerated FDA approval of Teliso-V (Emrelis) for adults with previously treated nonsquamous NSCLC and high c-MET protein overexpression, defined as ≥50% of tumor cells showing strong (3+) staining by an FDA-approved test.^25^^,^^26^ In a separate phase Ib trial, Teliso-V combined with erlotinib in *EGFR*-mutant, c-MET–positive NSCLC achieved an ORR of 32.1% (95% CI, 15.9-52.4), increasing to 52.6% in patients with high c-MET expression, suggesting potential utility in *EGFR* tyrosine kinase inhibitor (TKI) resistance settings.^23^

The safety profile of Teliso-V was consistent with other MMAE-containing ADCs. The most common treatment-related adverse events were peripheral sensory neuropathy (30%) and peripheral edema (16%), with grade ≥3 neuropathy occurring in 7% of patients.

While several investigational ADCs targeting c-MET—including ABBV-400, REGN5093-M114, and AZD9592—are in early-phase development,^22^^,^^27^^,^^28^ Teliso-V remains the only approved agent. Its success highlights c-MET overexpression as a therapeutically actionable and regulatory-recognized biomarker and affirms the broader potential of ADCs in biomarker-selected subsets of NSCLC.

#### TROP2

Trophoblast cell-surface antigen 2 (TROP2) is a transmembrane glycoprotein involved in cellular proliferation and broadly expressed in NSCLC. Several TROP2-directed ADCs are under development, with growing interest in biomarker-informed patient selection.^8^

Datopotamab deruxtecan (Dato-DXd) is an anti-TROP2 ADC conjugated to deruxtecan, a topoisomerase I inhibitor. In the phase 3 TROPION-Lung01 trial, Dato-DXd improved PFS over docetaxel in previously treated NSCLC, although OS did not differ significantly (hazard ratio [HR], 0.94; 95% CI, 0.78-1.14).^29^ Benefit was confined to nonsquamous NSCLC (median PFS 5.5 vs. 3.6 months; HR, 0.63; 95% CI, 0.51-0.79), while no improvement was observed in squamous disease (median PFS 2.8 vs. 3.9 months; HR, 1.41; 95% CI, 0.95-2.08). This striking divergence highlights histology-specific biology, as squamous tumors appear intrinsically less dependent on TROP2 signaling and may require rational combinations or alternative ADC designs to achieve benefit. Grade ≥3 TRAEs occurred in 25.6% of patients receiving Dato-DXd versus 42.1% with docetaxel. Among patients with *EGFR* mutations, the confirmed ORR was 41% (95% CI, 26-58), suggesting enhanced benefit here.

These findings were followed by TROPION-Lung05, a randomized phase 3 trial directly comparing Dato-DXd to docetaxel in patients with previously treated NSCLC.^30^ While the trial enrolled patients with various actionable mutations, the confirmed ORR was 35.8% (95% CI, 27.8-44.4) overall and 43.6% (95% CI, 32.4-55.3) in the *EGFR*-mutant subgroup. Given this differential efficacy, the FDA granted accelerated approval specifically for patients with *EGFR***-**mutant NSCLC in June 2025.^31^ The trial also met its primary endpoint of improved PFS and demonstrated a manageable safety profile. The most common TRAEs was stomatitis (any grade: 56.2%; grade ≥3: 9.5%). Adjudicated treatment-related ILD/pneumonitis occurred in 3.6% of patients, including 1 grade 5 event (0.7%).

A novel digital biomarker, the normalized membrane ratio (NMR), quantifies membrane localized versus total TROP2 and has been associated with improved ORR and PFS; given Dato-DXd’s reliance on membrane binding, NMR may better guide therapy in NSCLC.^32^

Further evidence supporting Dato-DXd in *EGFR*-mutant NSCLC was presented at the 2025 European Lung Cancer Conference (ELCC), where a phase 1b study evaluated its combination with osimertinib. Although early, the data showed promising tolerability and activity, reinforcing the rationale for ADC–TKI strategies in *EGFR*-driven disease.^33^

Sacituzumab govitecan (SG) is an anti-TROP2 ADC conjugated to SN-38. In the phase 3 EVOKE-1 trial, SG did not significantly improve OS over docetaxel in patients with previously treated NSCLC.^34^ However, In the phase 2 EVOKE-2 trial, SG was evaluated in combination with pembrolizumab as first-line therapy for advanced NSCLC, stratified by histology and programmed death-ligand 1 (PD-L1) tumor proportion score (TPS). Among patients with squamous histology, ORRs were 73% (95% CI, 39-94) in PD-L1 ≥ 50% (Cohort A, *n* = 11) and 54% (95% CI, 25-81) in PD-L1 < 50% (Cohort B, *n* = 13). In nonsquamous NSCLC, ORRs were 67% (95% CI, 41-87) in PD-L1 ≥ 50% (*n* = 18) and 37% (95% CI, 16-62) in PD-L1 < 50% (*n* = 19). Disease control rates (DCRs) exceeded 74% in all cohorts. Safety was consistent with known toxicities of SG and pembrolizumab; grade ≥3 treatment-emergent adverse events (TEAEs) occurring in ≥10% of patients included neutropenia (17%) and diarrhea (10%).^35^ These ORRs support the ongoing phase 3 EVOKE-3 trial of SG plus pembrolizumab versus pembrolizumab alone in PD-L1–high NSCLC.

Sacituzumab tirumotecan (SKB264) is a TROP2-directed ADC conjugated to a topoisomerase I inhibitor. In the phase 2 OptiTROP-Lung01 study, SKB264 was combined with KL-A167, a PD-1 inhibitor, in treatment-naïve patients with advanced NSCLC, including those with actionable genomic alterations.^36^ Patients received either Q3W or Q2W dosing, with ORRs of 48.6% (95% CI, 31.9-65.6) and 77.6% (95% CI, 64.7-87.5), respectively. Higher ORRs were observed among patients with elevated PD-L1 expression and squamous histology, though formal subgroup analyses are pending. Grade ≥3 TRAEs were dominated by cytopenias (notably neutropenia). A phase 3 trial (NCT06448312) is ongoing, evaluating SKB264 plus pembrolizumab versus pembrolizumab alone in PD-L1-high NSCLC.

SHRA1921(MK2870) is a novel TROP2-directed ADC conjugated to a topoisomerase I inhibitor via a cleavable tetrapeptide linker. In a pretreated NSCLC cohort, ORRs were 27.6% in nonsquamous and 15.8% in squamous histologies, with a median PFS of approximately 5.7 months.^37^ Grade 3 or 4 TRAEs occurred in 35.2% of patients, most commonly stomatitis (9.3%).

A global phase 3 trial (MSD007) is evaluating MK2870 plus pembrolizumab versus pembrolizumab alone in PD-L1–high NSCLC, positioning it directly against current standard of care.

One preclinical TROP2-targeted ADC, hIMB1636-LDP-AE—comprising a humanized anti-TROP2 antibody linked to the enediyne cytotoxin lidamycin—has shown potent antitumor activity and reduced myelotoxicity in early models, but has not yet entered clinical testing.^38^

Multiple TROP2-directed ADCs are advancing through late-phase development, and it is increasingly likely that more than one will ultimately receive regulatory approval in NSCLC. Efficacy appears to diverge by histology: Dato-DXd shows consistent benefit in nonsquamous tumors, while SG and SKB264 may retain activity in squamous subsets, particularly in PD-L1–enriched contexts. SHRA1921, while earlier in development, has reported modest efficacy in a pretreated cohort, with a phase 3 study planned in nonsquamous NSCLC. TROP2 IHC has not been used to guide patient selection in these trials, and its correlation with response remains unclear, underscoring the need for more refined biomarkers such as the NMR. Given differences in efficacy, safety, and patient selection, it is unlikely that a single TROP2-directed ADC will dominate; instead, multiple agents may find roles across subgroups.

#### HER3 and EGFR

Human epidermal growth factor receptor *3* (HER3) is frequently expressed in NSCLC and has become a promising target, particularly in *EGFR*-mutant tumors. *Patritumab deruxtecan* (HER3-DXd) is an anti-HER3 ADC conjugated to a topoisomerase I inhibitor. In the phase 2 HERTHENA-Lung01 trial, HER3-DXd achieved a confirmed ORR of 29.8% (95% CI, 23.9-36.2), with a median PFS of 5.5 months and median OS of 11.9 months.^39^ Responses were observed regardless of HER3 expression, including H-score 0, and were consistent across *EGFR*-dependent and -independent resistance. In patients with non-irradiated brain metastases, CNS ORR was 33.3%. Safety was manageable; grade ≥3 and ≥4 TEAEs occurred in 64.9% and 28.9% of patients, respectively. Although *HER3*-DXd demonstrated activity in the post-TKI, post-platinum setting—particularly in patients with brain metastases and regardless of *HER3* IHC status—development was discontinued in 2025 after HERTHENA-Lung02 failed to meet its endpoint (OS).

Bispecific ADCs targeting both HER3 and EGFR are also under investigation. In a Phase 1/2 study evaluating patients with TKI-pretreated, chemo-naiive NSCLC treated with izalontamab brengitecan (BL-B01D1), a bispecific HER3/EGFR ADC with a topoisomerase payload, ORR was 66% (cORR 56%) with a median PFS of 12.5 months, amongst patients with EGFR-mutated NSCLC. Frequent TRAEs included anemia (90.6%) and leukopenia (80.7%).^40^ I*zalontamab* (SI-B001), a bispecific HER3/EGFR ADC, was evaluated in a phase 2 study of EGFR/ALK wild-type NSCLC across 3 cohorts stratified by prior PD-1/L1 and platinum exposure, achieving an overall ORR of 31.3% (95% CI, 18.7-46.3) and a DCR of 77.1% (95% CI, 62.7-88.0); in Cohort B, AGA-negative patients showed an ORR of 50.0% (95% CI, 26.0-74.0). Toxicity was manageable.^41^ A phase 3 trial evaluating SI-B001 plus docetaxel in AGA-negative NSCLC is ongoing (NCT05943795).

Additional HER3- and EGFR-targeted ADCs in earlier development, such as MRG003 and M1231,^42^^,^^43^ are summarized in Table 2. Bispecific HER3/EGFR ADCs such as BL-B01D1 and SI-B001 have shown encouraging activity in NSCLC, with further studies needed to define durability and optimize patient selection, particularly in EGFR wild-type disease. These agents represent a promising class in both resistance-driven and immunotherapy-refractory settings, offering new avenues for targeted ADC therapy beyond canonical EGFR-TKI approaches.

**Table 2.** Selected emerging ADCs in NSCLC.

ADC name	Target	Development phase	Biomarker/selection	Notes
**Sacituzumab Tirumotecan (SKB264)**	*TROP2*	Phase 3	No selection	Ongoing phase 3 evaluating SKB264 + pembrolizumab versus pembrolizumab alone in PD-L1–high NSCLC.
**Tizetatug rezetecan (SHR-A1921)**	*TROP2*	Planned phase 3	No selection	Investigational Chinese ADC—planned for phase 3 evaluating pre-treated, non–squamous NSCLC
**Izalontamab brengitecan (BL-B01D1)**	*HER3/EGFR*	Phase 1/2	EGFR-mutant	ORR 66% (cORR 56%), mPFS 12.5 months; anemia 91%
**Izalontamab (SI-B001)**	*HER3/EGFR*	Phase 3	EGFR/ALK WT NSCLC post PD-1/L1 ± chemo	ORR 31%; *G* ≥ 3 myelosuppression = 17%, neutropenia = 15%
**Vobramitamab duocarmazine (MGC018)**	*B7-H3*	Phase 1	No selection	ORR 81%; 50% *G* ≥ 3 TRAEs (overall solid tumor cohort)
**Ifinatamab deruxtecan (DS-7300)**	*B7-H3*	Phase 1/2	Squamous NSCLC	ORR 40% (*n* = 5); 1 G5 ILD at 16 mg/kg (discontinued); low-grade ILD at 12 mg/kg, expansion ongoing
**Sigvotatug vedotin (SGN-B6A)**	*ITGB6*	Phase 1 (Parts C/D ongoing)	No selection	ORR 57%; *G* ≥ 3 TRAEs 61%; phase 3 trial ongoing with pembrolizumab in PD-L1–high, untreated NSCLC

ADC development in NSCLC continues to expand across a range of novel targets, including *TROP2, HER3, B7-H3, ITGB6, PTK7,* and *AXL*. This table highlights selected agents currently in phase 1-3 development with reported activity in NSCLC, focusing on biomarker context, preliminary efficacy, safety, and trial status. Agents are listed in order of discussion within the manuscript.

Abbreviations: ADC, antibody–drug conjugate; AGA, actionable genomic alteration; AXL, AXL receptor tyrosine kinase; *B7-H3*, B7 homolog 3 (CD276); CTCAE, common terminology criteria for adverse events; *EGFR*, epidermal growth factor receptor; *EGFRm*, EGFR-mutant; G5, grade 5 (fatal); *G* ≥ 3, grade ≥3; *HER3*, human epidermal growth factor receptor 3; ILD, interstitial lung disease; *ITGB6*, integrin beta 6; NSCLC, non–small cell lung cancer; ORR, objective response rate; PD-1/L1, programmed death-1 or ligand 1; PD-L1, programmed death-ligand 1; PFS, progression-free survival; *PTK7*, protein tyrosine kinase 7; TRAEs, treatment-related adverse events; WT, wild-type; taxane-naive, no prior taxane exposure; ALK, anaplastic lymphoma kinase.

#### Emerging ADC targets

Several novel ADC targets in NSCLC are under investigation. Integrin β6 (ITGB6) is a cell surface receptor implicated in tumor invasiveness. In an ongoing trial of sigvotatug vedotin (SV) plus pembrolizumab, among 7 efficacy-evaluable patients with PD-L1 TPS ≥1% NSCLC, the ORR was 57%, including 1 confirmed complete (CR) and 1 confirmed PR. Grade ≥3 TRAEs occurred in 61% of patients. A phase 3 trial is evaluating SV + pembrolizumab versus pembrolizumab alone in previously untreated, PD-L1–high advanced NSCLC (NCT06758401).^44^

B7 homolog 3 (B7-H3), an immune checkpoint molecule overexpressed in NSCLC, has been associated with poor prognosis and immune evasion. Two ADCs targeting B7-H3, MGC018 (duocarmycin payload) and DS-7300 (topoisomerase I inhibitor), have demonstrated early clinical activity. In a phase 1 study of MGC018, anti-tumor activity was observed in 13 of 16 evaluable patients, though 50% experienced grade ≥3 TRAEs.^45^ DS-7300 showed a 40% ORR (95% CI, 5-85) in patients with squamous NSCLC (*n* = 5); expansion cohorts are ongoing.^46^

AXL, a receptor tyrosine kinase linked to resistance to targeted therapy and immune evasion, was targeted by enapotamab vedotin (ORR 19%) before discontinuation^47^; another AXL-directed ADC, mecbotamab vedotin, remains under investigation in NSCLC (NCT04681131).

Carcinoembryonic antigen-related cell adhesion molecule 5 (CEACAM5; tusamitamab ravtansine) and sodium-dependent phosphate transport protein 2b (NaPi2b; lifastuzumab vedotin) have not advanced in NSCLC due to limited efficacy, while receptor tyrosine kinase-like orphan receptor 2 (ROR2; ozuriftamab vedotin) has been deprioritized with development shifting toward head and neck cancers.^48-50^ By contrast, tissue factor (TF; tisotumab vedotin) remains in active clinical development and is being evaluated in NSCLC (NCT03485209). HLX43, an anti–PD-L1 ADC, has successfully completed first-patient dosing in a phase 2 trial.^51^ Protein tyrosine kinase 7 (PTK7)–directed therapy has not advanced as a single-agent ADC following early trial failure; however, translational strategies such as the bispecific PTK7/EGFR ADC BCG017 are under investigation.^52^^,^^53^ Other emerging translational targets include folate receptor α (FRα; MORAb-202, PRO1184, STRO-002), transferrin receptor-1 (CD71; CX-2029), and activated leukocyte cell adhesion molecule (ALCAM; praluzatamab ravtansine).^54–56^

## Challenges and limitations

While ADCs have demonstrated therapeutic promise across hematologic and solid malignancies, several challenges remain in optimizing their development and delivery. Key barriers include the need for better predictive biomarkers, reducing treatment-related toxicity, overcoming drug resistance, and cost.

### Biomarker development

Unlike TKIs for which predictive biomarkers such as *EGFR* or *ALK* mutations are well-established, ADCs often lack validated, universally reliable selection tools. *HER2* and *HER3* mutations have demonstrated predictive value in NSCLC, particularly for agents like T-DXd and HER3-DXd, but ORRs remain lower than typically seen with matched TKIs.^2^^,^^57^  *HER2* amplification or IHC-based expression correlates inconsistently with clinical outcomes, while *c-MET* overexpression has shown therapeutic relevance in the context of Teliso-V, prompting regulatory recognition. This variability underscores that each ADC may rely on a distinct predictive paradigm, whether mutation, amplification, or surface expression, and that these relationships are not interchangeable across targets.

More sophisticated biomarker strategies are needed. For example, in TROP2-directed therapy, a novel computational biomarker—the NMR—has been associated with improved Dato-DXd response. In the *HER3* space, clinical benefit was observed regardless of HER3 IHC score, suggesting that even “low-expressing” tumors may respond to potent payloads. These examples highlight assay limitations and the potential for AI tools to improve selection.^32^^,^^57^^,^^58^

### Treatment-related ctoxicity

Toxicity remains a meaningful limitation of ADC therapy, particularly in patients with advanced NSCLC who may have reduced bone marrow reserve, reduced pulmonary function, or poor performance status. ADCs frequently cause cytopenias, gastrointestinal effects, and neuropathy. Organ-specific toxicities are also important: in DESTINY-Lung01, ILD occurred in 26% of patients receiving T-DXd, including 2 fatal cases.^14^ Dato-DXd, while better tolerated overall, has been associated with stomatitis and pneumonitis; in the TROPION-Lung05 trial, grade ≥3 TRAEs occurred in over 1-quarter of patients.^30^

Combinations may further increase toxicity risk while offering new therapeutic opportunities. In a phase 1b study of Dato-DXd plus osimertinib in *EGFR*-mutant NSCLC, the combination showed promising tolerability and antitumor activity, reinforcing the importance of dose optimization and early safety monitoring when integrating ADCs with existing standards of care.^33^

### Resistance mechanisms

Resistance to ADCs can arise through several mechanisms. Tumor cells may downregulate or alter the target antigen and/or limiting binding and internalization. Alternatively, increased efflux pump activity or impaired lysosomal processing may reduce payload delivery. These processes differ from canonical TKI resistance and remain less well characterized clinically. Preclinical studies also suggest roles for antigen shedding, linker instability, and bystander effects, though their integration into trial design is still early.^59^ Strategies to overcome resistance include bispecific ADCs, novel payloads, and combinations with checkpoint inhibitors or TKIs.

### Cost

ADCs are among the most expensive systemic cancer therapies. The complex manufacturing of antibody-linker-payload constructs, combined with frequent monitoring and toxicity management, contributes to high treatment costs. For example, SG has been associated with incremental costs exceeding $130 000 in metastatic breast cancer.^60^ Similar expenditures are likely in NSCLC. These costs may limit access, restrict formulary use, and raise cost-effectiveness concerns.

## Conclusion

ADCs have emerged as a promising therapeutic class in NSCLC, with efficacy across diverse molecular and clinical contexts. Successful integration into practice will require better biomarker strategies, toxicity management, and resistance mitigation. As each ADC may depend on distinct biological features—mutation, amplification, or protein expression—precision in patient selection is critical. We envision broad testing across targets (eg, HER2, TROP2, and c-MET) to identify patients most likely to benefit. Whether multiple distinct ADCs are needed will depend on the extent of clinical differentiation between agents, the risk of cross-resistance among ADCs with similar payloads, and whether testing infrastructure can support timely, multi-target biomarker selection in routine practice. With ongoing trials and advances in ADC design and biomarker assays, we anticipate their use in select first-line populations.

## Data Availability

No new data were generated or analyzed for this review article.
